# Novelty in Management of Traumatic Posterior Atlantoaxial Subluxation without Associated Fractures; A Case Report: Innovative Treatment for Atlantoaxial Subluxation

**DOI:** 10.31661/gmj.vi.3704

**Published:** 2025-08-04

**Authors:** Hamidreza Aghadoost, Hamed Yazdanpanah, Ghazaleh Salehabadi, Esmaeil Fakharian

**Affiliations:** ^1^ Department of Neurosurgery, School of Medicine, Kashan University of Medical Sciences, Kashan, Iran; ^2^ Department of Radiology, School of Medicine, Iran University of Medical Sciences, Tehran, Iran

**Keywords:** Posterior Atlantoaxial Subluxation, Fracture, Impactor

## Abstract

**Background:**

Background: Trauma is the main reason for Atlantoaxial subluxation in adults. The subluxation without fractures is extremely rare. Also, posterior atlantoaxial dislocation without odontoid fracture is extraordinarily uncommon and regularly causes deadly spinal cord injury. Based on the literature, there are few reports of approximately stressful posterior atlantoaxial dislocation, with incomplete quadriplegia related to a spinal cord injury.

**Case Presentation:**

Case Presentation: This report describes a 23-year-old Iranian man who was involved in a motor-to-barrier accident. He was hemodynamically stable with a Glasgow Coma Scale of 15/15 but exhibited cervical tenderness (C1-C2). A CT scan revealed a posterior C1-C2 subluxation, with the odontoid process anterior to the atlas and no fractures. Under general anesthesia, a fluoroscopy guide, and C-ARM imaging monitoring, flexion of the head and neck with traction was done. During the procedure, the odontoid process was locked in the inferior of the anterior C1 arch, Therefore, we decided to push back the odontoid process by using an Impactor through the retro pharynx. The maneuver was successful, and the odontoid was placed in its position.

**Conclusion:**

Conclusion: The stability of the atlantoaxial complex relies on its anatomy, which can be affected by trauma or congenital issues. Closed reduction followed by C1-C2 arthrodesis is the preferred treatment. Innovative methods, like using an impactor for closed reduction, need further study to improve outcomes. Surgeons must evaluate each case based on the patient’s specific situation.

## Introduction

Atlantoaxial subluxation is one of the most life-threatening causes in trauma cases. Neck tenderness, spinal cord compression as well as irreversible neurological deficits like cervical myopathy, paresis, and respiratory dysfunction are included as atlantoaxial subluxation complications [1]. Possessing posterior atlantoaxial dislocation without an affiliated atlas or odontoid fracture is extremely uncommon, and only a few cases are reported in the literature. Traumatic upper cervical spine injuries account for 20% of all acute cervical injury cases [2]. Traumatic atlantoaxial dislocation is uncommon and frequently manifests as anterior translational dislocation. High-velocity trauma usually is related to fracture. Therefore, after high-velocity trauma, atlantoaxial dislocation without any associated fractures is rare [3].

The literature on atlantoaxial dislocation highlights various cases and outcomes. Haralson (1969) reported a 30-year-old male with facial lacerations who underwent successful closed reduction and posterior fusion [4]. Sassard and Patzzkis (1974) documented a 20-year-old female and a 37-year-old male, both with facial injuries and no neurodeficits, achieving successful reductions [5][6]. Fox (1977) described a 65-year-old male with temporary paraplegia who required an anterior odontoidectomy and posterior fixation [7].

Jamshidi (1983) and Wong (1991) reported cases of a 22-year-old male and a 23-year-old male, respectively, both treated with posterior fusion [8][9]. Sud (2002) detailed a 38-year-old male with brachial plexus weakness who had a partial odontoidectomy after failed reduction [10]. Yoon (2003) mentioned a 22-year-old male with a subarachnoid hemorrhage treated with posterior fixation [11]. Neumann (2003) presented a 64-year-old male with facial injuries who benefited from closed reduction [12]. Chaudhary (2008) noted a 35-year-old female with facial lacerations who did not require surgery [13]. Lastly, Yong Xu (2015) discussed a 54-year-old male with partial lower extremity power, while Ghailane (2019) reported an 89-year-old male managed without operation. These cases reflect the diverse presentations and treatment outcomes associated with atlantoaxial dislocation [14][15].

Computed tomography is one of the recommended visualization modalities to provide data to decide on suitable management. In this report, we describe the surgery process of C1-C2 fixation in a rare case of posterior atlantoaxial subluxation without associated fractures.

## Case Presentation

**Table:** Table1. The subaxial cervical spine injury classification (SLIC) system and severity score

**Category**	**Parameter**	**Description**	**Points**
		Compression	1
		Burst	2
**1**	Injury morphology	Distraction	3
		Rotation/translation	4
		Intact	0
**2**	DLC ^*^ integrity	Suspected disruption	1
		Disruption	2
		Intact	0
		Nerve root injury	1
**3**	Neurological status	Complete cord injury	2
		Incomplete cord injury	3
		Persistence cord injury ^#^	+1
**Total Points **		**Management **	
1-3		Non-surgical	
4		Surgical or non-surgical	
5-10		Surgical	

^*^DLC: discoligamentous complex **#Neuro modifier:** continuous cord compression in the setting of a neurologic deficit

**Figure-1:**
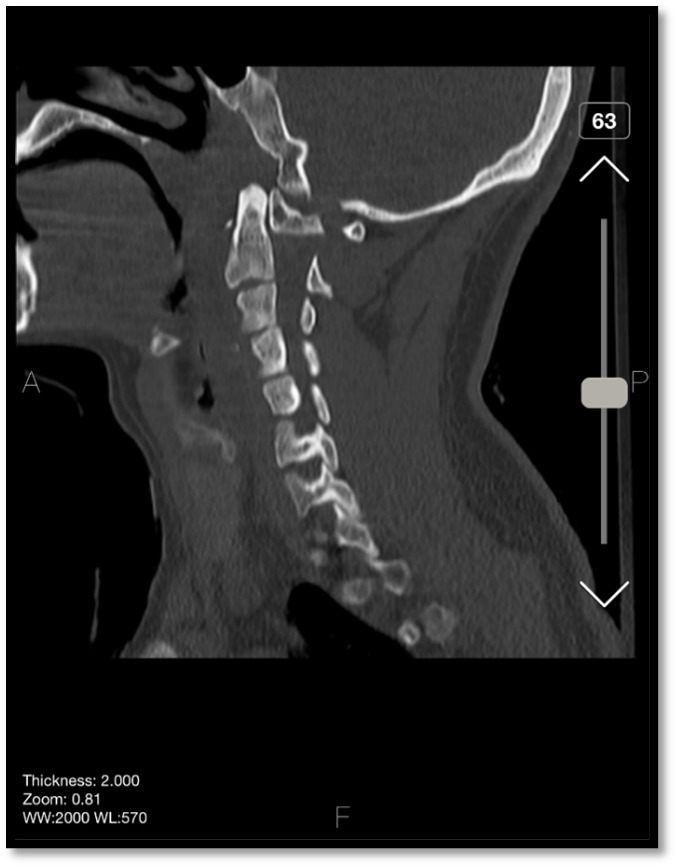
Figure 1. Pre-operative cervical CT scan shows posterior subluxation of the odontoid process lying in front of the anterior arch of the atlas, with no associated fractures or disruption of the spinolaminar line.

A 23-year-old Iranian gentleman was admitted to Shahid-Beheshti Hospital of Kashan and was hit by a Motor-to-Barrier Accident. He was fully conscious and oriented with a normal level of consciousness and was treated according to the Advanced Trauma Life Support Protocol. He was hemodynamically stable and experiencing mild respiratory distress due to cervical edema and tenderness. On examination, he had cervical tenderness (C1-C2) and a GCS of 15/15, pupils were symmetrical and reactive to light, and movement was normal in four extremities. There was no swelling or laceration in the scalp; however, tenderness in the neck was detected. The physical examination showed no neurological deficits, neither motor nor sensory. He had cervical motion and rotation limitations. His Deep tendon Reflexes were normal. A CT scan revealed a posterior subluxation of the odontoid process lay in front of the atlas anterior arch with no associated fractures and disruption of the spinolaminar line (Figure-1). No intracranial hemorrhage or midline shift was seen.

**Figure-2:**
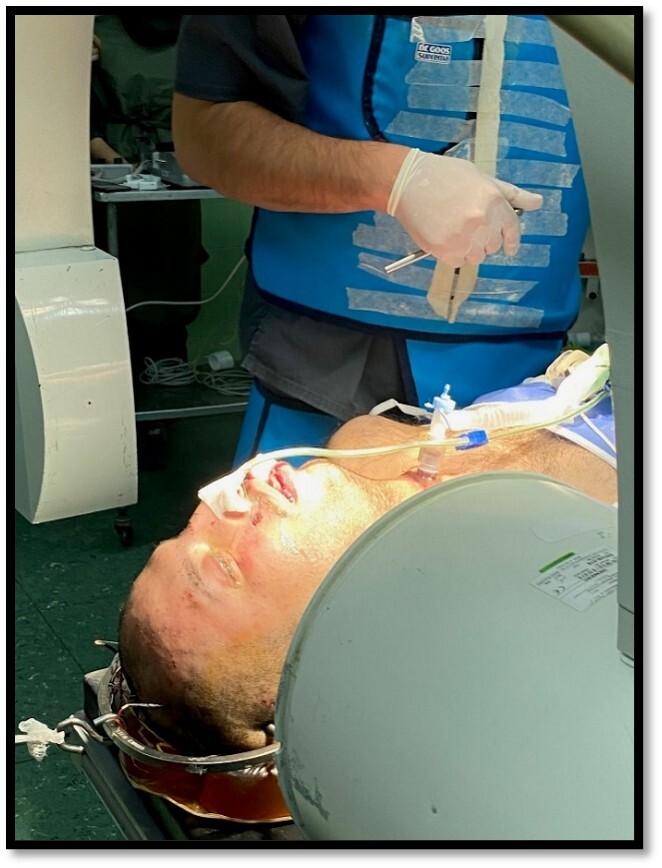
Figure 2. the patient under general anesthesia for an external reduction maneuver

**Figure-3:**
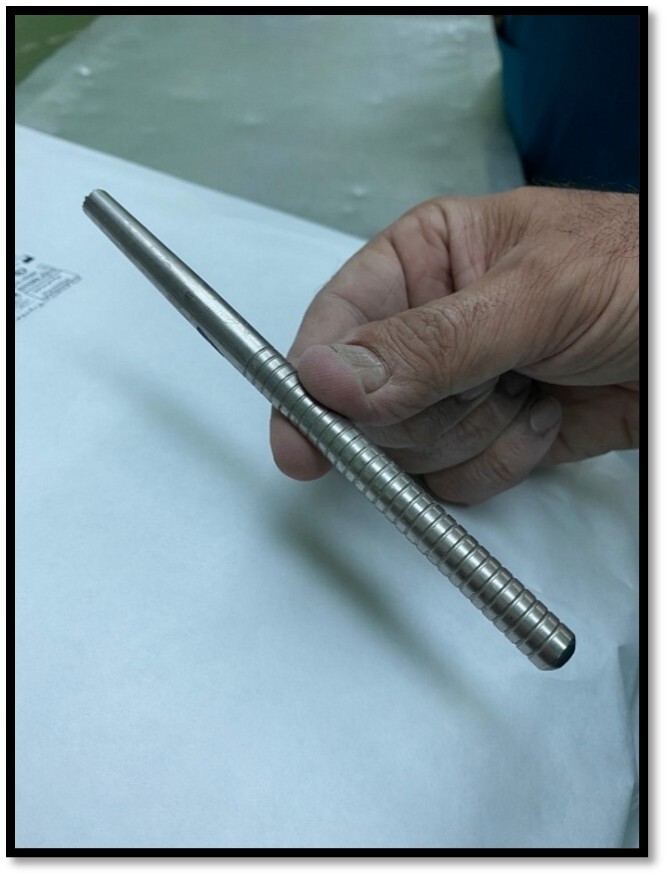
Figure 3. the impactor

**Figure-4:**
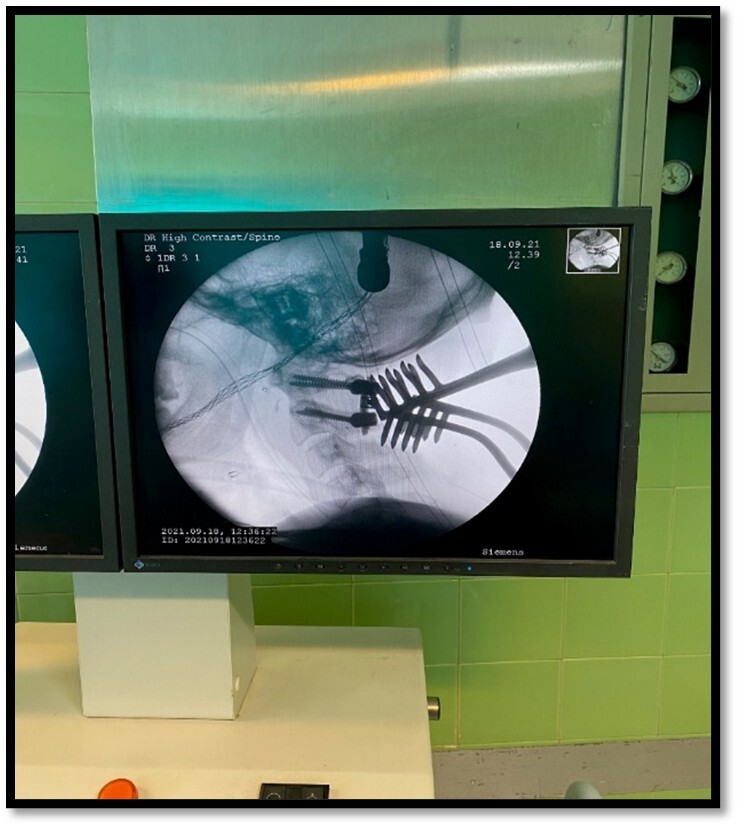
Figure 4. intraoperative fluoroscopic images

**Figure-5:**
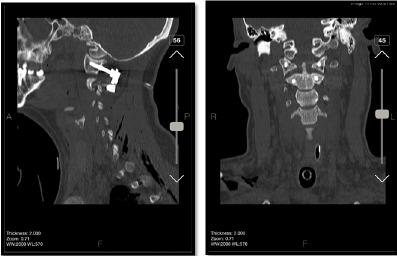
Figure 5. post-operative CT images show Lateral mass screw was applied for C1, and a pedicular measuring 24*8 mm in the right part of C2 and 26*4 mm in the left one were used. The fusion was applied to reduce ligament injuries

**Figure-6:**
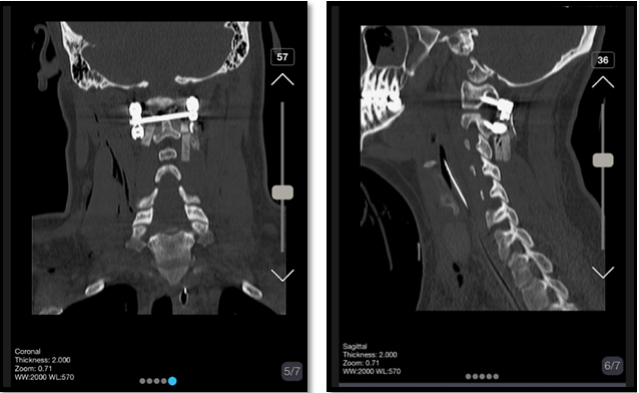
Figure 6. Cervical CT images after 1 week of surgery. The impactor showed to be placed appropriately, no sign of displacement was evident.

We used the collar to immobilize the patient’s neck due to cervical tenderness. Gardner-wells tongs were applied in the intensive care unit (ICU). Cervical traction within the neutral position employing a weight of 3 kg gradually increased to 5 kg was applied during immobilization for the first 48 hours. Despite the traction, no reduction was seen. He had respiratory distress because of the edema around his neck and respiratory tract. He underwent a tracheostomy due to his neck edema. A tracheostomy was applied since the intubation could fail because of the edema.

We utilized the Subaxial Cervical Spine Injury Classification (SLIC) system and severity score [16] to guide our treatment approach and predict the prognosis (Table-1). For the morphology category, we assigned a score of 4 due to the translation morphology of the injury. Unfortunately, we could not conduct an MRI to assess the ligamentous injury accurately because the patient’s critical status required urgent management. All distraction and translation bony injury morphologies indicate a disruption of the disco-ligamentous complex. Therefore, we assigned a score of 1 for the disco-ligamentous complex (DLC) integrity, indicating suspected disruption. The neurological status of the patient was intact, resulting in a score of 0 for that category.

The total score was calculated to be 5, which indicates that the patient requires surgical treatment. Subsequently, the patient was transferred to the operating room.

Under general anesthesia, the patient was placed in the supine position. Under fluoroscopy guide and C-ARM imaging monitoring, several times of closed reductions were attempted through gradual manual cranial traction from flexion to extension (Figure-2). To sum up the surgery process; we can say that three phases were done, first of all, the flexion of the head and neck with traction was done, then neutral position applied when the odontoid process was near the posterior wall of the anterior C1 arch. During the process, the odontoid process was locked in the inferior of the anterior C1 arch. Therefore, in the 3rd phase, we decided to push back the odontoid process by using an impactor (Figure-3) through the retro pharynx. The maneuver was successful, and the odontoid was placed in its position. All phases were done under neuromonitoring. Then, He was positioned from supine to prone to fix the C1 and C2 vertebrae. We exposed the axis and atlas surgically via a posterior approach. The attempts to relocate the normal axis-atlas alignment to fuse the facet joints failed. The lateral mass screw was applied for C1, and pedicular 24*8 mm in the right part of C2 and 26*8 mm in the left one was used. The fusion was applied to reduce the ligament injuries (Figure-4and5).

The day after the surgery, the patient reported experiencing cervical pain and nausea. A physical examination revealed no neurological deficits, and the surgery site appeared clean with no signs of infection. However, mild cervical edema was present, so we closely monitored the patient’s respiratory status, which remained stable with an oxygen saturation of 97%, and no signs of respiratory distress were observed. The patient was advised to drink fluids, consume soft foods, and start moving out of bed after 24 hours post-surgery. The drains were removed within 48 hours.

The patient was discharged with a cervical collar after three days. He returned for a follow-up visit after one week, during which we conducted a cervical CT scan (Figure-6). In the CT scan, the impactor showed to be placed appropriately, no sign of displacement was evident. At this point, tracheostomy was removed, the patient reported significant improvement and no longer experienced cervical pain.

After one month, the collar was removed, and the patient regained the ability to move his neck in various directions without limitations in motion or rotation. He was able to return to work. We continued to follow up with the patient for six months, during which no complications, such as impaction displacement, associated fractures, or vascular issues arose.

## Discussion

The atlantoaxial complex’s intrinsic stability is primarily provided by the interlocking articular process and the odontoid process, which is interlocked in an osteo-ligamentous ring formed ventrally by the atlas anterior arch and posteriorly by the transverse ligament. The majority of the atlantoaxial subluxations are caused by odontoid fracture or rupture of the transverse atlantal ligament. These dislocations are usually anterior due to the relatively weak ligamentous structure of the ring in the posterior section [15]. To the best of our knowledge, the incidence of anterior or posterior atlantoaxial subluxation without related fractures is very rare in literature.

Atlantoaxial dislocation is frequently caused by trauma, tumors, or congenital malformations in the upper cervical region and is a complicated cranial-cervical junction disorder. These dislocations can cause spinal cord or medulla compression, resulting in limb numbness and weakness, sphincter dysfunction, disrupted circulation, and respiration center dysfunction. According to reports, the mortality rate of traumatic atlantoaxial dislocation is 60-80% [17]. Therefore, choosing proper management techniques for these patients is critical. As the gold standard treatment, the current scientific literature recommends closed reduction followed by C1-C2 arthrodesis [18]. More cases are expected, but as most of these patients lead to death, the available case reports are minimal. Haralson and Boyd presented the first case of posterior atlantoaxial subluxation in 1969 and proposed hyperextension with variable amounts of distraction as the probable mechanism of posterior dislocation without odontoid fracture [4].

Impactors are usually applied in orthopedic surgical procedures to insert the bone graft. Based on our knowledge, there was no report of applying an impactor for external maneuver for a closed reduction in atlantoaxial subluxation. We used an impactor in this case to push back the odontoid process, and it was a successful attempt. It seems that further studies are needed to examine this new procedure to decrease failed closed reduction. As the current scientific literature recommends, closed reduction followed by C1-C2 arthrodesis is gold standard management; using a finger or tools such as an impactor we used to push back the odontoid process in its proper position was the critical approach.

The high-velocity accidents are usually related to atlantoaxial dislocation. Therefore, a systematic trauma assessment must be applied to decrease the mortality and morbidity rates [6].

In the field of surgical treatment, it’s crucial to emphasize the importance of personalized care tailored to each patient’s unique situation. Management strategies cannot be universally applied; they must be adapted to fit individual needs. Patients presenting with similar conditions may have different underlying factors that influence their treatment. Surgeons should take a comprehensive view of each case, considering variables such as medical history, current health status, and specific circumstances. This nuanced approach ensures that treatment plans are effective and align with the patient’s overall well-being.

The case report exemplifies how thoughtful intraoperative decisions can be pivotal in saving lives. Such decisions require a well-informed surgeon to carefully weigh possible approaches and adapt quickly to the patient’s needs during surgery. By fostering a deep understanding of various techniques, surgeons can perform safe manual reductions under neuromonitoring, enhancing both safety and patient outcomes.

## Conclusion

Consequently, correction of atlantoaxial dislocation is a complicated procedure, with a high associated mortality and morbidity rate and potentially severe neurologic deficits. The stability of the atlantoaxial complex is primarily dependent on its anatomical structures, which can be compromised by trauma or congenital anomalies. Nowadays, closed reduction before C1-C2 arthrodesis is used as the gold standard of treatment.

New approaches, such as using an impactor for closed reduction, deserve further investigation to improve patient outcomes. It is necessary for surgeons to evaluate each case individually and according to the specific conditions of the patient. Knowing different management methods along with proper decision-making during surgery and neuromonitoring can be effective in optimizing the surgical results and ultimately improving the patient’s survival and quality of life.

## Conflict of Interest

The authors had no conflicts of interest.
